# Weight trajectories through infancy and childhood and risk of non-alcoholic fatty liver disease in adolescence: The ALSPAC study

**DOI:** 10.1016/j.jhep.2014.04.018

**Published:** 2014-09

**Authors:** Emma L. Anderson, Laura D. Howe, Abigail Fraser, Mark P. Callaway, Naveed Sattar, Chris Day, Kate Tilling, Debbie A. Lawlor

**Affiliations:** 1 MRC Integrative Epidemiology Unit at the University of Bristol, Bristol, UK; 2 School of Social and Community Medicine, University of Bristol, Bristol, UK; 3 University Hospitals Bristol NHS Foundation Trust, UK; 4 Institute of Cardiovascular & Medical Sciences, BHF Glasgow Cardiovascular Research Centre, Faculty of Medicine, University of Glasgow, UK; 5 Institute of Cellular Medicine, Faculty of Medical Sciences, Newcastle University, UK

**Keywords:** NAFLD, Non-alcoholic fatty liver disease, ALT, Alanine aminotransferase, AST, Aspartate aminotransferase, GGT, Gamma-glutamyl transferase, ALSPAC, Avon Longitudinal Study of Parents and Children, AUDIT, Alcohol Use Disorders Identification Tests, ARFI, Acoustic radiation force impulse-imaging, DXA, Dual-energy X-ray absorptiometry, BMI, Body mass index, Infant, Childhood, Growth, Obesity, BMI, NAFLD, Fatty liver

## Abstract

**Background & Aims:**

Adiposity is a key risk factor for NAFLD. Few studies have examined prospective associations of infant and childhood adiposity with subsequent NAFLD risk. We examined associations of weight-for-height trajectories from birth to age 10 with liver outcomes in adolescence, and assessed the extent to which associations are mediated through fat mass at the time of outcome assessment.

**Methods:**

Individual trajectories of weight and height were estimated for participants in the Avon Longitudinal Study of Parents and Children using random-effects linear-spline models. Associations of birthweight (adjusted for birth length) and weight change (adjusted for length/height change) from 0–3 months, 3 months–1 y, 1–3 y, 3–7 y, and 7–10 y with ultrasound scan (USS) determined liver fat and stiffness, and serum alanine aminotransferase (ALT), aspartate aminotransferase (AST), and gamma-glutamyl transferase (GGT) at mean age 17.8 y were assessed with linear and logistic regressions. Mediation by concurrent fat mass was assessed with adjustment for fat mass at mean age 17.8 y.

**Results:**

Birth weight was positively associated with liver stiffness and negatively with ALT and AST. Weight change from birth to 1 y was not associated with outcomes. Weight change from 1–3 y, 3–7 y, and 7–10 y was consistently positively associated with USS and blood-based liver outcomes. Adjusting for fat mass at mean age 17.8 y attenuated associations toward the null, suggesting associations are largely mediated by concurrent body fatness.

**Conclusions:**

Greater rates of weight-for-height change between 1 y and 10 y are consistently associated with adverse liver outcomes in adolescence. These associations are largely mediated through concurrent fatness.

## Introduction

Non-alcoholic fatty liver disease (NAFLD) is one of the most common causes of chronic liver disease in children and adolescents in the developed world [1], [2], and is associated with fibrosis, insulin resistance, and dyslipidaemia, independently of total body fat [3]. Greater adiposity is a key risk factor for NAFLD; previous studies have reported NAFLD prevalences of up to 80% in obese children [4], as well as cross-sectional associations of BMI in childhood and adolescence with NAFLD [5], [6], [7]. Prospective studies assessing associations of adiposity, or rates of change in adiposity throughout infancy and childhood with NAFLD at a later age are lacking. Such studies are important for establishing the age at which these associations emerge. Furthermore, understanding associations of change in adiposity across infancy and childhood with later NAFLD is important for establishing whether there are sensitive periods for the development of NAFLD.

Previous studies suggest that infancy is a sensitive period in the relationship between increasing weight and later cardiometabolic risk [8], [9], [10]. However, more recent evidence from the ALSPAC cohort suggests that weight gain most proximal to the cardiometabolic outcomes is more strongly associated with these outcomes compared with weight gain earlier in life [11], [12]. One previous study reported positive associations of conditional weight growth from ages 0–11 years with NAFLD outcomes, with associations from age 2 years attenuating after adjustment for adult BMI [13]. Another study reported a higher prevalence of NAFLD in children born small for gestational age who experienced rapid catch-up growth in the first year of life, compared to appropriate for gestational age controls [14].

The aims of this study were: (i) to assess associations of BMI at different time-points between birth and age 10 years with markers markers of NAFLD (USS determined liver fat and stiffness [a marker of fibrosis] and blood-based indicators of liver function; alanine aminotransferase (ALT), aspartate aminotransferase (AST) and gamma-glutamyl transferase (GGT)) at mean age 17.8 years, (ii) to assess associations of trajectories of weight change, adjusted for height change, from birth to age 10 years with markers of adolescent NAFLD and (iii) to examine the extent to which any observed associations in change in adiposity between birth and age 10 are mediated through fat mass at the time outcomes were assessed.

## Materials and methods

### Study population

**Fig. 1:**
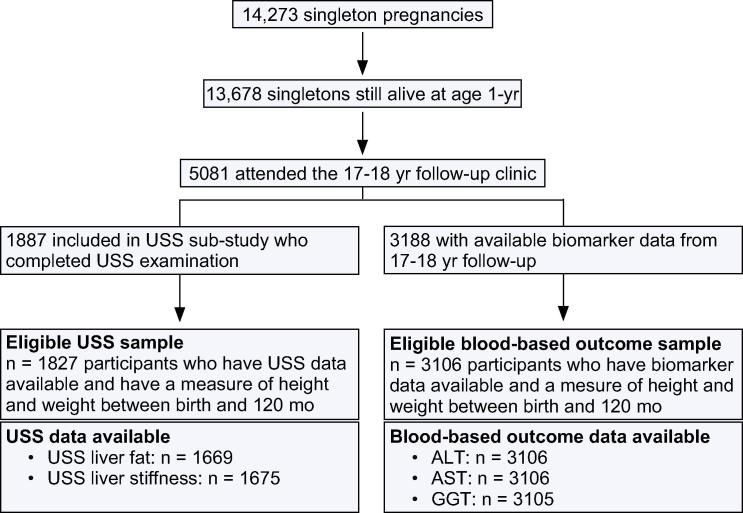
**Participant flow through the study.** Participants were excluded if they had no weight or height measures between birth and 10 years or they had harmful alcohol consumption.

ALSPAC is a population-based, prospective birth cohort from southwest England (www.alspac.bris.ac.uk). Full details of the study have been published previously [15], [16]. Ethical approval for the study was obtained from the ALSPAC Ethics and Law Committee and Local Research Ethics Committees. Five-thousand and eighty-one singletons attended a 17–18 year follow-up assessment (mean age 17.8 years) and of these, 3188 [62.7%] had data available for blood-based indicators of liver function including ALT, AST, and/or GGT. A liver USS sub-study was undertaken on a sub-group of participants attending the 17–18 year follow-up. Sub-study participants were a quasi-random group of those attending the 17–18 year follow-up in that the sub-study began 10 months after the general follow-up and only participants who attended the general follow-up assessment on a day that one of the trained sonographers was working were included. A total of 1887 sub-study participants completed the USS examination (Fig. 1). Sub-study participants are representative of the ALSPAC cohort [3]. Participants’ alcohol consumption was assessed by questionnaires at the 16–17 year and 17–18 year follow-ups, using the Alcohol Use Disorders Identification Tests (AUDIT) questionnaire [17]. Participants answered 10 questions about their alcohol consumption, and from their responses, a score between 0 and 20 was derived. A score over 16 is classified as harmful alcohol consumption [17]. Thirteen participants who completed the USS examination and 29 participants with blood-based liver outcomes scored greater than 16 at both ages and were excluded from this study. No participants had a known history of jaundice or hepatitis, were taking medications or receiving treatment that would indicate they had hepatic disease, or were taking medication known to influence liver function.

### Assessment of outcomes

Participants attending the 17–18 year clinic in the morning were instructed to fast overnight or for a minimum of 6-h, for those attending after lunch.

#### USS assessment of liver fat and stiffness

##### Assessment of USS liver fat and stiffness

The protocol for USS assessment in ALSPAC has been published previously [3]. Briefly, upper abdominal USS was completed by one of four trained sonographers using a Siemens Acuson S2000 USS system. Echogenicity, our marker of liver fat, was assessed during deep inspiration and recorded as present, absent or uncertain according to established protocols [18], [19]. A longitudinal image, in the sagittal plane, with juxta position of the right lobe of the liver and the right kidney was viewed and echogenicity determined by comparison between the liver and kidney. Levels of agreement in identifying echogenicity between the four sonographers was 98% or greater, both immediately after training and at 6-month intervals throughout data collection. Acoustic radiation force impulse-imaging (ARFI) of the right lobe of the liver was used to measure liver stiffness (our main indicator of liver stiffness), using standard protocols [20], [21]. ARFI, measured as shear velocity in meters/second (m/s), was assessed six times with a gap of at least 1 min between each measurement. The highest and lowest of these measurements were excluded and the Siemens Acuson S2000 system produced a mean of the remaining four measurements. If this mean was greater than four m/s, a further six measurements were taken from the left lobe. When both right and left lobe values were available, the lowest mean of the two has been used in analyses.

##### Assessment of blood-based indicators of liver function

Fasting blood samples were immediately spun and frozen at −80 °C. Measurements were assayed shortly (3–9 months) after samples were taken with no previous freeze-thaw cycles. All assays were completed in the same laboratory at the University of Glasgow. ALT, GGT, and AST were measured by automated analyser with enzymatic methods. We defined those with a value of >30 U/L ALT as having abnormally high ALT levels, using the most common blood-based indicator and the threshold that has been used previously in adolescent/childhood populations [22]. All inter- and intra-coefficients of variation for these blood-based assays were <5%.

##### Assessment of exposures and covariables

Length/height and weight data are available from several sources in ALSPAC at birth and throughout infancy and childhood. Full details of these measurements are provided in the Supplementary data. Potential confounders considered in this analyses are as follows: age at outcome assessment, gender, gestational age, breastfeeding at age 3 months, parity, maternal age at the delivery, maternal pre-pregnancy smoking status and alcohol consumption, and household social class. Fat mass and height at age 17.8 years were considered as potential mediators. At the 17–18 year follow-up visit, a Lunar prodigy narrow fan beam densitometer was used to perform a whole body dual-energy X-ray absorptiometry (DXA) scan, from which total fat mass was measured. Height was measured to the nearest 0.1 cm using a Harpenden stadiometer with the participant unshod. The assessment of all potential confounding variables is described in detail in the Supplementary data.

#### Statistical analyses

##### Estimating weight and height change trajectories from birth to 10 years

Individual weight and length/height trajectories from birth to 10 years were estimated using linear spline multilevel models, fitted in MLwiN version 2.25 using the Stata (*StataCorp*, College Station, *Texas*) command ‘runmlwin’ [23], [24]. They were estimated for all participants with at least one available weight and height measure between these ages, under a missing at random (MAR) assumption. In our study, birth weight (in kilograms) and length (in centimeters), and the rate of linear weight change (in kilograms/year) and length/height change (in centimeters/year) from birth to 3 months, 3 months to 1 year, 1 to 3 years, 3 years to 7 years, and 7 years to 10 years were estimated [25]. This pre-pubertal age range was selected to avoid the difficulty of being able to separate an effect of adiposity change from an effect of puberty, which has major influences on weight/adiposity change, and to provide a clear separation between the exposures and outcomes. Full details of the weight and length/height models have been published previously [25] and are available on request.

##### Infant and childhood body mass index (BMI)

The trajectories described above were used to predict each participant’s height and weight at ages 3 months and 1, 3, 7, and 10 years. BMI was calculated at these ages as predicted weight divided by predicted height in meters-squared. BMI was standardised by sex to internal z-scores.

##### Associations of BMI and weight-for-height change trajectories with liver outcomes

ALT, AST, GGT, and liver stiffness were positively skewed and their natural logged values were used in all linear regression analyses. Coefficients from regression models including these logged variables as outcomes were back transformed and are interpreted as the percentage change in each outcome per standard deviation increase in the exposure. Logistic or linear regression models examined associations of infant and childhood BMI and weight-for-height trajectories with measures of liver health at mean age 17.8 years.

Associations of BMI with liver outcomes were examined using the following models: (1) unadjusted and (2) adjusted for all potential confounders. Associations of weight trajectories with the liver outcomes were adjusted for length/height trajectories to ensure any observed associations were due to greater relative adiposity, rather than greater weight as a result of greater height. Associations of weight trajectories with the liver outcomes were examined in the following models: (1) unadjusted; (2) adjusted for length or length/height change in the same period as the weight or weight change exposure (e.g., for associations where weight change from 3–7 years is the exposure, height change from 3–7 years is adjusted for); (3) additionally adjusted for all potential confounders. Breastfeeding is not adjusted for when birth weight is the exposure variable as it occurs after the exposure and therefore cannot confound these associations; (4) same as model 3 with additional adjustment for potential confounding by all previous weight and length change; and (5) same as model 4 with additional adjustment for DXA determined fat mass, height and height-squared, assessed at the same time as the liver outcomes. Model 5 examines potential mediation of associations of weight-for-height change throughout infancy and childhood with liver outcomes by concurrent total body fat. Likelihood ratio tests were used to check for gender interactions in all models.

##### Dealing with missing data and additional analyses

Thirty four percent (n = 619/1827) of eligible participants with USS data, and 25% (n = 790/3106) of eligible participants with blood-based liver outcomes had missing data for some potential confounders and outcomes (missing data for any one characteristic varied from 0.01% to 13%; Supplementary Tables 1 and 3). To minimise selection bias and increase efficiency, multivariate multiple imputation was used to impute missing data for potential confounders and outcomes for eligible participants in the two separate samples. Full details of this procedure are provided in the Supplementary data. Details of a series of sensitivity analyses that were conducted to verify our statistical model assumptions and test the robustness of our findings are also provided in the Supplementary data.

## Results

Prevalence of USS fatty liver was 2.5% in males (n = 17/685) and 2.6% in females (n = 26/958). ALT was elevated (>30 U/L) in 10.4% of males (n = 156/1495) and 4.5% of females (n = 73/1611). Table 1 provides a description of various characteristics the USS and blood-based liver outcomes imputed datasets. A previous publication provides a comparison of these characteristics between those who do and do not have USS identified liver fat, and details the prevalence of fibrosis in the subgroup with USS data [3]. Supplementary Tables 1 and 2 compare distributions of participant characteristics in the imputed and observed data.

**Table 1:** **A description of characteristics for the USS and blood-based liver outcomes datasets.**

n.a., not available.

### Associations of infant and childhood BMI with liver outcomes

Point estimates for associations between BMI and both the USS and blood-based liver outcomes increased with age at measurement (Table 2, Table 3). For example, odds of having USS liver fat per standard deviation increase in BMI increased from 1.31 (95%CI: 0.97–1.75) at age 3 to 2.28 (95%CI: 1.78–2.92) at age 10, and the percentage change in ALT per standard deviation increase in BMI increased from +3% (95%CI: +1% ± 4%) at age 3 to +8% (95%CI: +6% ± 9%) at age 10.

**Table 2:** **Associations of infant and childhood BMI with USS liver fat and liver stiffness in the imputed data (n = 1827).**

Coefficients are per 1 standard deviation (SD) increase in BMI at ages 3 months and 1, 3, 7, and 10 years. The SDs for BMI at each age are as follows: BMI at age 3 months = 1.43 kg/m^2^; BMI at age 1 year = 1.40 kg/m^2^; BMI at age 3 years = 1.11 kg/m^2^; BMI at age 7 years = 1.58 kg/m^2^; BMI at age 10 years = 2.64 kg/m^2^.

^a^Adjusted for age of the study child at the outcome assessment and potential confounding by gender, gestational age, breastfeeding at age 3 months, parity, maternal age at the birth of the study child, maternal pre-pregnancy smoking status and alcohol consumption and household social class.

n.a., not available.

**Table 3:** **Associations of infant and childhood BMI with ALT, AST, and GGT in the imputed data (n = 3106).**

Coefficients are per 1 standard deviation (SD) increase in BMI at ages 3 months and 1, 3, 7, and 10 years. The SDs for BMI at each age are as follows: BMI at age 3 months = 1.42 kg/m^2^; BMI at age 1 year = 1.37 kg/m^2^; BMI at age 3 years = 1.09 kg/m^2^; BMI at age 7 years = 1.51 kg/m^2^; BMI at age 10 years = 2.52 kg/m^2^.

^a^Adjusted for age of the study child at the outcome assessment and potential confounding by gender, gestational age, breastfeeding at age 3 months, parity, maternal age at the birth of the study child, maternal pre-pregnancy smoking status and alcohol consumption and household social class.

### Associations of weight-for-height trajectories from birth to 10 years with liver outcomes

**Fig. 2:**
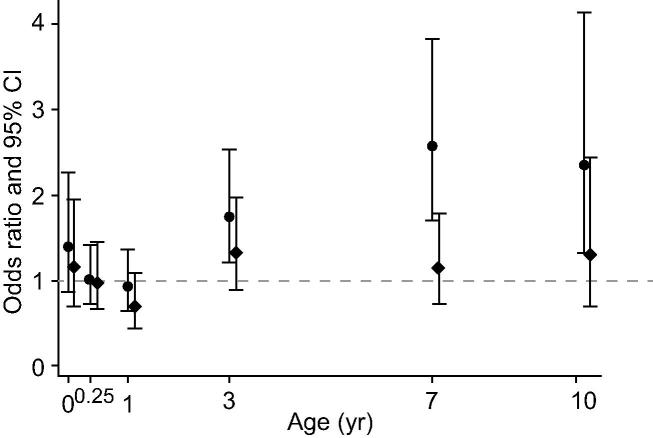
**Associations of weight-for height change from birth to 10 years with USS liver fat in the main confounder adjusted model (circular dots) and the model additionally adjusting for potential mediation by concurrent fat mass (diamond dots), in the imputed data (n = 1827).** Coefficients are odds ratios for USS liver fat per 1 standard deviation (SD) increase in birth weight or rate of weight change in each age period of childhood (markers placed at the end of each period of childhood).

**Fig. 3:**
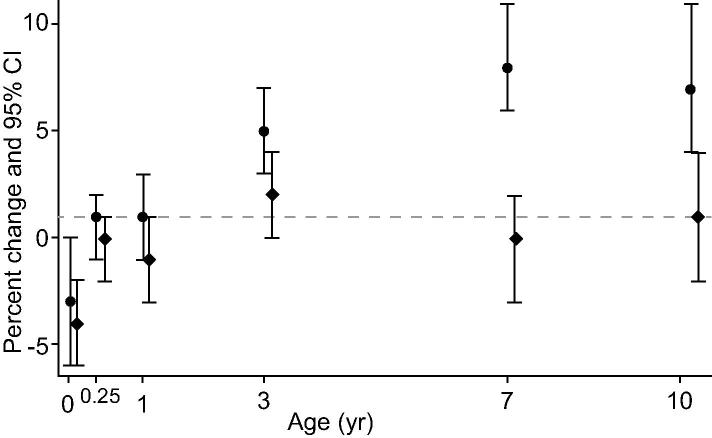
**Associations of weight-for-height change from birth to 10 years with ALT in the main confounder adjusted model (circular dots) and the model additionally adjusting for potential mediation by concurrent fat mass (diamond dots), in the imputed data (n = 3106).** Coefficients are per 1 standard deviation (SD) increase in birth weight or in the rate of weight change in each age period of childhood (markers placed at the end of each period of childhood).

Birth weight-for-length was positively associated with liver stiffness but not with liver fat, and negatively associated with ALT and AST. There was no evidence for associations of weight-for-length change from birth to age 1 with any of the liver outcomes. Weight-for-height change after age 1 year was positively associated with all liver outcomes, except that weight-for-height change from ages 7–10 was not associated with AST. Associations of weight-for-height trajectories with USS liver fat and ALT in the main confounder adjusted model (model 4) are presented in Fig. 2, Fig. 3, respectively. Associations of weight-for-height trajectories with all other USS and blood-based liver outcomes (in all models) are presented in Supplementary Tables 3 and 4.

### Mediation by fat mass at the time of outcome assessment

After additional adjustment for total body fat mass at the time liver outcomes were assessed, all associations attenuated toward the null, though some associations remained. Results of this model (model 5) are presented for liver fat and ALT in Fig. 2, Fig. 3 respectively.

### Gender differences

There was statistical evidence of a gender interaction for associations of weight-for-length/height change in some periods with some, but not all outcomes. However, there was no consistent pattern and in all cases, the direction of the associations for males and females was the same, with only the magnitudes differing (results available on request).

### Additional analyses

Results were similar when restricted to participants with complete data on all variables included in the analysis, when restricted to participants that had at least 2 measures of weight and height from birth to 10 years, and when restricted to participants with a measure of weight and height in each age period defined by our linear spline model. Results were also very similar after additional adjustment for AUDIT scores, and when using ponderal index instead of BMI. We found no evidence of an interaction with birthweight (i.e., no evidence that associations of weight change from birth to 10 years with the liver outcomes differ for those in the lowest tertile of birth weight compared to those in the upper two tertiles). Results from all additional analyses are available on request.

## Discussion

In this study we assessed prospective associations of BMI at different ages in infancy and childhood, and of trajectories of weight-for-height change from birth to age 10 years, with measures of liver health in adolescence. BMI at ages 7 and 10 years were consistently positively associated with all liver outcomes, whilst associations of BMI at younger ages differed by age and outcome. Using weight-for-height trajectories as exposures, we found that rates of weight-for-height change between ages 1 and 10 years were consistently positively associated with both USS and blood-based outcomes, whereas associations of birth weight-for-length and weight-for-length change in the first year of life were generally weak and inconsistent. Thus, our results suggest that greater adiposity gain (assessed by weight-for-height change) in childhood, particularly from age 3 years of age, and higher BMI at age 7 and 10 years are importantly and consistently associated with a range of different USS and blood-based markers of liver damage in adolescence. Interventions to limit adiposity gain from early in childhood are likely to be important to prevent future adolescent, and potentially adult, adiposity related liver disease.

Although infancy has previously been suggested as a sensitive period for associations between greater adiposity and later adverse cardiometabolic risk factors, our findings do not support this; we found there were no consistent associations of BMI in infancy with the liver outcomes. Our finding that rates of change in adiposity between 1 and 10 years are consistently positively associated with the liver outcomes, with increasing coefficient magnitudes throughout childhood, suggests there is no sensitive period when greater rates of adiposity change is particularly detrimental and that greater increases in adiposity between ages 1 and 10 years might increase risk of NAFLD in adolescence. From a public health perspective, this study highlights the need to control weight/fat gain at young ages in order to limit total body fat and the risk of NAFLD in adolescence.

### Comparisons with other studies

Cross-sectional studies have shown that greater BMI (and other measures of adiposity) in childhood and adolescence is associated with greater risk of NAFLD assessed by USS [26], blood-based indicators [5], [6], [7], [26], biopsy [27], and at post-mortem [1], [28]. Consistent with a previous study of adult women, (mean age 68 years, n = 2106) we found greater birth weight to be associated with lower ALT and AST levels [29]. Other studies of both adults and children have reported no association between birth weight and ALT [5], [30]. Our finding that birth weight is positively associated with USS liver fat and fibrosis is inconsistent with the inverse association with ALT and AST, and with what we might expect from the developmental origins literature, which suggests poor intrauterine nutrition results in a lower birth weight and adverse development of the liver and other organs [31], [32]. Studies using more direct assessments of liver pathology have reported a strong and graded inverse relationship between birth weight and length and mortality from cirrhosis [33], and associations of small for gestational age with increased risk of subsequent NAFLD [14], [34]. Another study found several measures of birth and childhood body size to be inversely related to NAFLD outcomes in a group of 1587 individuals from the Helsinki Birth Cohort. Similar to our findings, the same study also reported positive associations of conditional weight growth from 2 to 11 years with NAFLD outcomes, however, once adult BMI was adjusted for, coefficients became negative [13].

### Strengths and limitations

Our study was undertaken in a large contemporary cohort of adolescents and we were able to adjust for a wide range of potential confounders. The extent of repeat height and weight measurements in our study means we were able to assess whether there are any specific periods of development where rates of adiposity change are particularly strongly associated with indicators of NAFLD for the continuously measured outcome. However, we acknowledge that power for the binary outcome of USS NAFLD was limited due to the small number of cases. That said, results were generally consistent for all outcomes. Advantages of using multilevel models to obtain trajectories of weight and length/height have been described in detail in a previous publication [25].

USS is not the ‘gold standard’ for identifying NAFLD, however, it is neither feasible nor ethical to undertake liver biopsies in large cohorts of healthy people. Studies have shown USS to accurately identify moderate to severe steatosis compared with liver biopsy in adults and children [35], [36] and this has been summarised in a systematic review and meta-analysis [37]. Thus, our prevalence estimate for NAFLD may reflect the moderate to severe end of the spectrum of this disease. The ARFI measure of liver stiffness used in our study is a relatively new measure, but has been validated in a small number of clinical studies [38], [39]. In models where we adjust for potential mediation by fat mass at the time of outcome assessment, there is potential for collider bias to occur [40]. This means that adjusting for a potential mediator can introduce residual confounding between the exposure and the outcome, if there are unadjusted confounders of the mediator-outcome relationship. In this study, the most plausible confounders of the fat mass-NAFLD association are diet and physical activity. We were unable to adjust for these, because they were not measured at age 17. Evidence from a recent study suggests that, in practice, this generally does not result in considerable bias [41]. The MAR assumption underlying our linear-spline models is likely to hold in our example; results were similar in the complete-case analysis and when analyses were repeated on individuals with two or more weight and length/height measures or at least one measure per linear-spline period. This cohort is largely white European (+96%) and we cannot assume results necessarily generalize to other ethnic groups. We were unable to examine change in other measures of adiposity (such as DXA determined total (or trunkcal) fat mass or waist circumference) with liver outcomes in this study as these measurements were not undertaken in infancy and early childhood in ALSPAC. However, our earlier publication shows the relationships of BMI, DXA determined total and trunkcal fat mass with hepatic outcomes are similar [42]. In this study we used AUDIT to assess alcohol consumption in all participants. Although AUDIT has been validated in adolescents, it is possible that participants may misreport their alcohol consumption and that some NAFLD cases might be alcohol-related. However, this is unlikely, firstly because alcohol-induced steatosis requires high levels of alcohol consumption for a prolonged period of time, which is less likely to occur at age 17–18 years than at older ages, secondly because we used a validated tool to remove those with harmful consumption over the 12 months prior to liver assessment, and additionally adjusted for the continuous AUDIT scores with very little change to the results, and finally because our previous publication shows the majority of NAFLD cases to be at the upper end of the fat mass distribution [3].

## Conclusions

In conclusion, our results suggest that greater adiposity gain from 1 year of age is associated with adverse levels of a range of USS and blood-based markers of liver disease, and that these associations are likely mediated through concurrent adiposity at the time of outcome assessment. Since our study was conducted in healthy participants with no known evidence of liver disease and in whom those with persistent high levels of alcohol consumption over the previous 12 months were removed, our study supports initiatives aimed at promoting healthy growth and adiposity gain from early childhood to prevent NAFLD in adolescence/young adulthood. Future randomised controlled trials of interventions for limiting adiposity gain in childhood would also be valuable in assessing the effect on NAFLD risk in adolescence.

## Financial support

The research leading to these results has received funding from the UK Medical Research Council (G0801456), the 10.13039/501100000274British Heart Foundation (PG/11/33/28794), and the European Union’s Seventh Framework Programme (FP7/2007–2013) under grant agreement n° HEALTH-F2-2009-241762 for the project FLIP. The UK Medical Research Council and Wellcome Trust (092731), together with the University of Bristol, provide core support for the ALSPAC study. DAL, AF and LDH work in a unit that receives funding from the UK Medical Research Council and EA’s studentship is funded by that grant. AF and LDH are funded by UK Medical Research Council Post-doctoral research fellowships (G0701594, and G1002375, respectively).

## Conflict of interest

The authors who have taken part in this study declared that they do not have anything to disclose regarding funding or conflict of interest with respect to this manuscript.

## Authors’ contributions

ELA undertook all analyses and wrote the first draft of the paper; LDH developed the analysis plan, supervised analyses and contributed to writing early drafts of the paper as part of the core writing group; AF obtained funds, developed the original study aims and contributed to writing early drafts of the paper as part of the core writing group; MPC obtained funds, supervised collection of the ultrasound scan data and commented on the final drafts of the paper; NS obtained funds, supervised all laboratory analyses and commented on the final drafts of the paper; CD obtained funds and commented on final drafts of the paper; KT developed the analysis plan and supervised analyses; DAL obtained funds, developed the original study aims and the analysis plan and contributed to writing early drafts of the paper as part of the core writing group. ELA and DAL act as guarantors for the paper.
