# Supplementary material for: Weight trajectories through infancy and childhood and risk of non-alcoholic fatty liver disease in adolescence: The ALSPAC study
Source: J Hepatol. 2014 Sep;61(3):626–32. doi: 10.1016/j.jhep.2014.04.018 (PMC4139262; doi:10.1016/j.jhep.2014.04.018)
Supplement: Supplementary data [file mmc1.pdf]

Supplementary data

Supplementary materials and methods

Assessment of weight and length/height in ALSPAC

Length/height and weight data are available from several sources in ALSPAC. Crown-heel birth length was measured by ALSPAC staff who visited newborns soon after birth (median 1 day, range 1-14 days), using a Harpenden Neonatometer (Holtain Ltd). Birth weight was extracted from medical records. Length/height and weight measurements from birth to five years were extracted from health visitor records, which form part of standard childcare in the UK. Participants had up to four measurements taken, on average at six weeks, 10, 21, and 48 months of age, which have been previously shown to have good accuracy [1]. A random 10% of the cohort also have length/height and weight measurements from research clinics between four months and five years of age. From age seven years upwards, all children were invited to annual clinics. Full details of the measurement procedures at the research clinics are in the online supplement. Across all ages, parent-reported child heights and weights were also obtained from questionnaires.

Between four months and five years, crown-heel length for children aged 4 to 25 months was measured using a Harpenden Neonatometer and from 25 months onwards standing height was measured using a Leicester Height Measure. Weight was measured using Fereday 100kg combined scale (4 month clinic), Soenhle scale or Seca scale model 724 (8 month clinic), Seca 724 or Seca 835 (12 month clinic), Seca 835 (18 months onwards). From age seven years onwards, all children were invited to annual clinics, at which standing height was measured to the last complete millimetre using the Harpenden Stadiometer and weight was measured to the nearest 0.1kg using the Tanita Body Fat Analyser (Model TBF 305).

Assessment of potential confounders

Participants' age at outcome assessment was calculated from the date of attendance and the participants' birth date. Maternal age at birth of the child and the child's gestational age and sex were obtained from obstetric records. Questionnaires administered to mothers early in pregnancy asked about parity, pre-pregnancy smoking status and alcohol consumption. Information on breastfeeding was assessed repeatedly during infancy by postal questionnaires, and from the responses, a variable was derived with the categories: "exclusive breastfeeding at age 3 months", "non-exclusive/stopped breast feeding by age 3 months" and "never breastfed". Based on questionnaire responses, the highest parental occupation was used to allocate participants to family social class groups using the 1991 British Office of Population and Census Statistics (OPCS) classification.

Details of the multiple imputation procedure

All participants included in this study had a weight and length/height trajectory from birth to 120 months, however 28.7% (n = 525/1827) of eligible participants with USS data, and 19.2% (n = 597/3106) of eligible participants with biomarker data had missing data for some potential confounders and outcomes. To minimise selection bias and increase efficiency, multivariable multiple imputation was used to impute missing data for potential confounders and outcomes, as well as potential predictors of the missing data, for eligible participants in the two separate samples. Regression switching was used in Stata, as described by Royston [2]. Twenty cycles of regression switching were carried out and 20 imputation datasets were generated. We found evidence of a gender interaction for associations of weight gain after 12 months with liver stiffness (all p values for interaction <0.05), and of weight gain after 36 months with ALT and GGT (all p values for interaction <0.05) in the complete case analyses (i.e., including participants with no missing data), thus, we carried out the multivariate multiple imputations

separately for males and females and appended the two sets of datasets together to allow us to assess interactions in the multiple imputation datasets. The multiple multivariate imputation approach creates a specified number of copies of the data (in our case, 20 copies) in which missing values are imputed by chained equations, with an appropriate level of randomness. The main results presented in this paper on the multiple imputation datasets are obtained by averaging the results from each of these 20 datasets using Rubin's rules. In this procedure, the standard errors for any regression coefficients (used to calculate p-values and 95% confidence intervals) take account of the uncertainty in the imputations as well as uncertainty in the estimate. The analyses based on these multivariate imputations all include data from the 1827 eligible participants in the USS dataset and the 3106 participants in the biomarker dataset. We also repeated analyses including only those participants with complete data on all variables used in our analyses, i.e., with no missing data (n = 1281 for the USS dataset and n = 2467 for the biomarker dataset). Results from the complete case analyses were very similar to those found based on the multiple imputation datasets. Multiple imputation results are presented in Supplementary Tables 3 and 4 below**.** Complete case analysis results are available on request.

Additional analyses

To verify the MAR assumption of the multilevel models and assess potential bias caused by missing data, analyses including the weight trajectories as exposures were repeated after restricting to participants with (i) at least two measures of weight and length/height from birth to 10 years (n = 1822 in the USS dataset and n = 3075 in the blood-based liver outcomes dataset) and (ii) at least one measure of weight and length/height in each of the age periods defined by our linear-splines (n = 1249 in the USS dataset and n = 2093 in the blood-based liver outcomes dataset). Analyses were also repeated for participants with complete data for all variables included in the analysis (i.e., with no missing data). In additional analyses, we adjusted for

AUDIT scores (in addition to removing those with persistent high scores over the year prior to the 17-18y follow-up). AUDIT scores from the time of the outcome assessments (mean age 17.8y) were used if available (80% and 83% of eligible participants in the USS and blood-based liver outcomes datasets, respectively). AUDIT scores assessed in the previous year (mean age 16.7y) were used if the score from the time of the outcome assessments was not available (11% and 10% of eligible participants in the USS and blood-based liver outcomes datasets, respectively). The remaining 9% and 7% of AUDIT scores for eligible participants in the USS and blood-based liver outcomes datasets, respectively, were imputed as described above. As BMI in infants under age 2 years may not be a useful measure of adiposity, analyses were repeated using Ponderal Index as the exposure, instead of BMI. Additional analyses were not repeated for associations of infant and childhood BMI with the liver outcomes, as BMI variables were derived from the height and weight trajectories. We tested for evidence of an interaction with birth weight by including a binary interaction term in all regression models, comparing participants in the lowest tertile of birth weight to those in the upper two tertiles of birth weight.

Supplementary Tables

Supplementary Table 1. Distribution of variables in the observed (with no missing) and imputation USS datasets.

**Imputed Variables**		**Distribution variables in the observed USS dataset (with no missing) n = 1208**		**Distribution variables in the imputation dataset for eligible participants with USS data n = 1827**		
**Continuous variables**		**Mean (SE)/Median (IQR)**	**Range**	**% Imputed data**	**Mean (SE)/Median (IQR)**	**Range**
Median USS liver stiffness, m/s (IQR)		1.18 (1.07, 1.32)	0.69, 3.73	8	1.19 (1.08, 1.32)	0.67, 4.55
Median gestational age at birth, weeks (IQR)		40 (39, 41)	32, 44	4	40 (39, 41)	26, 44
Mean Maternal age, years (SE)		29.90 (0.13)	17.00, 43.67	3	29.70 (0.12)	16.21, 46.54
Median fat mass at mean age 17.8y, kg (IQR)		16.71 (11.48, 23.45)	1.44, 65.56	4	16.95 (11.49, 23.88)	1.43, 89.86
Mean height at mean age 17.8y, cm (SE)		171.01 (0.27)	146.50, 197.70	3	170.66 (0.22)	144.6, 201.5
**Categorical variables**		**Percent (n)**			**Percent**	
USS liver fat	Yes	2.11		9	2.94	
	No	97.89			97.06	
Breastfeeding at 3 months	Never	14.49		12	15.12	
	Non exclusive/stopped	48.43			49.24	
	Exclusive	37.09			35.64	
Parity	0	49.26		8	48.59	
	1	35.68			34.67	
	2+	15.07			16.75	
Head of household social class	Manual	13.19		13	13.85	

	Non-manual	86.81		86.15
Mother's pre-pregnancy smoking	None	87.28	11	85.95
	1-6 per day	4.68		5.22
	>6 per day	8.04		8.83
Mothers pre-pregnancy alcohol consumption	<1 glass/wk	40.98	7	42.92
	1+ glass/wk	46.84		44.52
	1+ glass/day	12.18		12.55
Alcohol consumption in the year prior to outcome assessment*	Hazardous	-	10	33.18
	Harmful	-		5.27

SD, standard deviation.

*In additional analyses on the USS imputed dataset, AUDIT scores were adjusted for. AUDIT scores from the time of the outcome assessments (at mean age 17.8y) were used if available (80% of eligible participants in the USS dataset). AUDIT scores assessed in the previous year (mean age 16.7y) were used if the score from the time of the outcome assessments was not available (11% of eligible participants in the USS dataset). The remaining 9% of AUDIT scores for eligible participants in the USS dataset were imputed.

Supplementary Table 2. Distribution of variables in the observed (with no missing) and imputation biomarker datasets.

**Imputed Variables**		**Distribution variables in the observed biomarker dataset (with no missing) n = 2316**		**Distribution variables in the imputed dataset for eligible participants with blood based biomarker data n = 3106**		
**Continuous variables**		**Mean (SE)/Median (IQR)**	**Range**	**% Imputed data**	**Mean (SE)/Median (IQR)**	**Range**
Median ALT, U/L (IQR)		14.90 (11.90, 19.50)	0.20, 315.90	0	15.1 (11.9, 19.6)	0.20, 315.9
Median AST, U/L (IQR)		19.60 (16.80, 23.10)	7.30, 213.60	0	19.6 (16.8, 23.2)	7.3, 213.6
Median GGT, U/L (IQR)		16 (13, 21)	6, 453	0.03	16.0 (13.0, 21.0)	6.0, 453
Median gestational age at birth, weeks (IQR)		40 (39, 41)	30, 43	4	40 (39, 41)	27, 45
Mean Maternal age, years (SE)		29.98 (0.09)	17, 43.67	4	29.80 (0.08)	16.31, 43.94
Median fat mass at mean age 17.8y, kg (IQR)		15.96 (10.24, 22.45)	1.68, 65.30	4	16.03 (10.26, 22.74)	1.44, 162.08
Mean height at mean age 17.8y, cm (SE)		172.21 (0.19)	146.50, 208	3	172.02 (0.17)	144.6, 208
**Categorical variables**					**Percent**	
Breastfeeding at 3 months	Never	13.21		13	13.22	
	Non-exclusive/stopped	47.63			48.53	
	Exclusive	39.16			38.25	
Parity	0	47.71		7	47.51	
	1	36.20			35.53	
	2+	16.09			16.96	
Head of household social class	Manual	11.84		11	13.07	
	Non-manual	88.16			86.93	
	None	88.77		12	87.51	

Mother's pre-pregnancy smoking	1-6 per day	3.81		4.14
	>6 per day	7.42		8.35
Mothers pre-pregnancy alcohol consumption	<1 glass/wk	41.22	6	42.27
	1+ glass/wk	46.33		44.99
	1+ glass/day	12.44		12.74
Alcohol consumption in the year prior to outcome assessment*	Hazardous	-	7	34.82
	Harmful	-		4.78

SD, standard deviation.

*AUDIT scores from the time of the outcome assessments (at mean age 17.8y) were used if available (83% of eligible participants in the biomarker dataset). AUDIT scores assessed in the previous year (mean age 16.7y) were used if the score from the time of the outcome assessments was not available (10% of eligible participants in the biomarker dataset). The remaining 7% of AUDIT scores for eligible participants in the biomarker dataset were imputed.

Supplementary Table 3. Associations of weight-for-height change from birth to 120 months with USS liver fat, liver stiffness and liver volume in the imputed dataset of eligible participants with USS data (n = 1827).

	USS Liver Fat			USS Liver Fibrosis		
	**OR**	**95% CI**	**P**	**% Change**	**95% CI**	**P**
**Model 1**						
Birth weight	1.12	(0.81,1.56)	0.50	+1%	(0%,+2%)	0.06
Weight-for-height change 0-3 months	0.97	(0.72,1.29)	0.82	+1%	(0%,+2%)	0.14
Weight-for-height change 3 months-1 year	0.92	(0.68,1.24)	0.58	0%	(-1%,+1%)	0.65
Weight-for-height change 1-3 years	1.72	(1.33,2.24)	<0.01	+1%	(0%,+2%)	0.01
Weight-for-height change 3-7 years	1.96	(1.58,2.43)	<0.01	+3%	(+2%,+4%)	<0.01
Weight-for-height change 7-10 years	2.12	(1.71,2.63)	<0.01	+3%	(+2%,+4%)	<0.01
**Model 2**						
Birth weight	1.36	(0.85,2.16)	0.20	+1%	(0%,+3%)	0.07
Weight-for-height change 0-3 months	0.98	(0.72,1.35)	0.92	0%	(0%,+1%)	0.33
Weight-for-height change 3 months-1 year	0.88	(0.61,1.28)	0.51	-1%	(-2%,0%)	0.26
Weight-for-height change 1-3 years	1.69	(1.19,2.39)	<0.01	+2%	(+1%,+3%)	<0.01
Weight-for-height change 3-7 years	2.38	(1.72,3.30)	<0.01	+4%	(+3%,+6%)	<0.01
Weight-for-height change 7-10 years	2.38	(1.85,3.05)	<0.01	+3%	(+2%,+4%)	<0.01
**Model 3**						
Birth weight	1.40	(0.87,2.26)	0.17	+2%	(0%,+3%)	0.02
Weight-for-height change 0-3 months	0.98	(0.71,1.36)	0.92	0%	(-1%,+1%)	0.43
Weight-for-height change 3 months-1 year	0.91	(0.63,1.31)	0.61	-1%	(-2%,0%)	0.27
Weight-for-height change 1-3 years	1.74	(1.23,2.47)	<0.01	+2%	(+1%,+3%)	<0.01
Weight-for-height change 3-7 years	2.49	(1.75,3.54)	<0.01	+4%	(+3%,+6%)	<0.01
Weight-for-height change 7-10 years	2.52	(1.90,3.33)	<0.01	+3%	(+2%,+5%)	<0.01
**Model 4**						
Birth weight	1.40	(0.87,2.26)	0.17	+2%	(0%,+3%)	0.02
Weight-for-height change 0-3 months	1.02	(0.73,1.41)	0.91	+1%	(0%,+2%)	0.21
Weight-for-height change 3 months-1 year	0.93	(0.63,1.37)	0.72	-1%	(-2%,0%)	0.24
Weight-for-height change 1-3 years	1.75	(1.21,2.53)	<0.01	+2%	(0%,+3%)	0.01
Weight-for-height change 3-7 years	2.57	(1.72,3.84)	<0.01	+4%	(+3%,+6%)	<0.01

Weight-for-height change 7-10 years	2.35	(1.33,4.16)	<0.01	+3%	(+1%,+5%)	0.01
**Model 5**						
Birth weight	1.17	(0.70,1.95)	0.55	+2%	(0%,+3%)	0.04
Weight-for-height change 0-3 months	0.97	(0.66,1.43)	0.88	0%	(-1%,+1%)	0.55
Weight-for-height change 3 months-1 year	0.70	(0.45,1.08)	0.11	-1%	(-2%,0%)	0.05
Weight-for-height change 1-3 years	1.33	(0.90,1.97)	0.16	+1%	(-1%,+2%)	0.25
Weight-for-height change 3-7 years	1.15	(0.74,1.78)	0.54	+3%	(+1%,+4%)	<0.01
Weight-for-height change 7-10 years	1.30	(0.70,2.44)	0.41	+2%	(0%,+4%)	0.14

The SDs for birth weight and the weight change periods are as follows: birth weight = 0.48kg; weight-for-height gain 0-3 months = 0.19kg/month; weight-for-height gain 3 months-1 year = 0.09kg/month; weight-for-height gain 1-3 years = 0.03kg/month; weight-for-height gain 3-7 years = 0.05kg/month; weight-for-height gain from 7-10 years = 0.11kg/month. Model 1 is unadjusted. Model 2 adjusts for length or length/height change in the same period as the weight or weight change exposure. Model 3 is additionally adjusted for age of the study child at the outcome assessment and potential confounding by gender, gestational age, breastfeeding at age 3 months, parity, maternal age at the birth of the study child, maternal pre-pregnancy smoking status and alcohol consumption and household social class. Breastfeeding is not adjusted for when birth weight is the exposure variable.

Table 3. Associations of weight-for-height change from birth to 120 months with ALT and AST in the imputed dataset of eligible participants with biomarker data (n = 3106).

	ALT			AST			GGT		
	**% Change**	**95% CI**	**P**	**% Change**	**95% CI**	**P**	**% Change**	**95% CI**	**P**
**Model 1**									
Birth weight	-1%	(-3%, 0%)	0.10	-1%	(-2%, 0%)	0.03	-1%	(-3%, 0%)	0.08
Weight-for-height change 0-3 months	+1%	(0%, +3%)	0.11	0%	(-1%, +1%)	0.85	+2%	(+1%, +3%)	0.01
Weight-for-height change 3 months-1 year	+2%	(0%, +3%)	0.04	+1%	(-1%, +2%)	0.33	+2%	(+1%, +4%)	<0.01
Weight-for-height change 1-3 years	+4%	(+2%, +6%)	<0.01	+1%	(0%, +2%)	0.04	+3%	(+2%, +5%)	<0.01
Weight-for-height change 3-7 years	+7%	(+5%, +8%)	<0.01	+1%	(0%, +2%)	0.05	+5%	(+4%, +7%)	<0.01
Weight-for-height change 7-10 years	+8%	(+6%, +9%)	<0.01	+1%	(0%, +2%)	0.10	+6%	(+4%, +7%)	<0.01
**Model 2**									
Birth weight	-3%	(-5%, 0%)	0.02	-2%	(-4%,-1%)	0.01	-2%	(-4%, 0%)	0.11
Weight-for-height change 0-3 months	+1%	(-1%, +3%)	0.22	0%	(-1%, +1%)	0.64	+2%	(0%, +3%)	0.02
Weight-for-height change 3 months-1 year	+1%	(0%, +3%)	0.13	+1%	(0%, +2%)	0.12	+1%	(0%, +3%)	0.08
Weight-for-height change 1-3 years	+4%	(+2%, +6%)	<0.01	+2%	(0%, +3%)	0.02	+2%	(+1%, +4%)	0.01
Weight-for-height change 3-7 years	+8%	(+6%, +11%)	<0.01	+2%	(0%, +3%)	0.02	+7%	(+5%, +9%)	<0.01
Weight-for-height change 7-10 years	+8%	(+6%, +10%)	<0.01	+1%	(0%, +3%)	0.03	+7%	(+5%, +8%)	<0.01
**Model 3**									
Birth weight	-3%	(-6%, 0%)	0.04	-2%	(-4%,-1%)	<0.01	-2%	(-4%, 0%)	0.09
Weight-for-height change 0-3 months	+1%	(-1%, +2%)	0.38	0%	(-1%, +1%)	0.73	+2%	(0%, +3%)	0.04
Weight-for-height change 3 months-1 year	+1%	(0%, +3%)	0.14	+1%	(0%, +2%)	0.19	+1%	(0%, +3%)	0.10

Weight-for-height change 1-3 years	+4%	(+2%, +6%)	<0.01	+2%	(0%, +3%)	0.02	+2%	(+1%, +4%)	<0.01
Weight-for-height change 3-7 years	+8%	(+6%, +10%)	<0.01	+2%	(0%, +3%)	0.03	+7%	(+5%, +9%)	<0.01
Weight-for-height change 7-10 years	+8%	(+6%, +10%)	<0.01	+1%	(0%, +2%)	0.04	+7%	(+5%, +8%)	<0.01
**Model 4**									
Birth weight	-3%	(-6%, 0%)	0.04	-2%	(-4%,-1%)	<0.01	-2%	(-4%, 0%)	0.09
Weight-for-height change 0-3 months	+1%	(-1%, +2%)	0.56	0%	(-1%, +1%)	0.95	+1%	(0%, +3%)	0.08
Weight-for-height change 3 months-1 year	+1%	(-1%, +3%)	0.27	+1%	(-1%, +2%)	0.29	+1%	(0%, +3%)	0.16
Weight-for-height change 1-3 years	+5%	(+3%, +7%)	<0.01	+2%	(+1%, +3%)	<0.01	+3%	(+2%, +5%)	<0.01
Weight-for-height change 3-7 years	+8%	(+6%, +11%)	<0.01	+2%	(0%, +3%)	0.03	+6%	(+4%, +9%)	<0.01
Weight-for-height change 7-10 years	+7%	(+4%, +11%)	<0.01	0%	(-2%, +2%)	0.87	+5%	(+3%, +8%)	<0.01
**Model 5**									
Birth weight	-4%	(-6%,-2%)	<0.01	-3%	(-4%,-1%)	<0.01	-3%	(-5%,-1%)	0.01
Weight-for-height change 0-3 months	0%	(-2%, +1%)	0.68	0%	(-1%, +1%)	0.68	+1%	(-1%, +2%)	0.37
Weight-for-height change 3 months-1 year	-1%	(-3%, +1%)	0.26	0%	(-1%, +1%)	0.72	-1%	(-2%, +1%)	0.52
Weight-for-height change 1-3 years	+2%	(0%, +4%)	0.05	+2%	(0%, +3%)	0.03	+1%	(-1%, +3%)	0.30
Weight-for-height change 3-7 years	0%	(-3%, +2%)	0.86	0%	(-1%, +2%)	0.77	-1%	(-3%, +2%)	0.54
Weight-for-height change 7-10 years	+1%	(-2%, +4%)	0.52	-1%	(-3%, +1%)	0.32	0%	(-3%, +3%)	0.99

Coefficients are per 1 standard deviation (SD) increase in weight at age 3 months or per SD increase in the rate of weight change in each age period defined by the linear spline model. The SDs for birth weight and the weight change periods are as follows: birth weight = 0.49kg; weight-for-height gain 0-3 months = 0.49kg/month; weight-for-height gain 3 months-1 year = 0.09kg/month; weight-for-height gain 1-3 years = 0.03kg/month; weight-for-height gain 3-7 years = 0.05kg/month; weight-for-height gain from 7-10 years = 0.10kg/month. Model 1 is unadjusted. Model 2 adjusts for length or length/height change in the same period as the weight or weight change exposure. Model 3 is additionally adjusted for age of the study child at the outcome assessment and potential confounding by gender, gestational age, breastfeeding at age 3 months, parity, maternal age at the birth of the study child, maternal pre-pregnancy smoking status and alcohol consumption and household social class. Breastfeeding is not adjusted for when birth weight is the exposure variable.

Supplementary references

[1] Howe LD, Tilling K, Lawlor DA. Accuracy of height and weight data from child health records. Archives of Disease in Childhood 2009 Dec 1;94(12):950-954.
[2] Royston P. Multiple imputation of missing values. Stata J 2004;4:227-241.
